# Isopropyl *N*-[1′-(methoxy­carbon­yl)ferro­cen­yl]carbamate–ethyl *N*-[1′-(methoxy­carbon­yl)ferrocen­yl]carbamate (0.6/0.4)

**DOI:** 10.1107/S1600536809008204

**Published:** 2009-03-14

**Authors:** Anas Lataifeh, Heinz-Bernhard Kraatz, Michael C. Jennings

**Affiliations:** a Department of Chemistry, The University of Western Ontario, London, Ontario, Canada N6A 5B7

## Abstract

Herein we report the crystal structure and synthesis of two cocrystallized complexes, [Fe(C_7_H_7_O_2_)(C_9_H_12_NO_2_)]_0.6_[Fe(C_7_H_7_O_2_)(C_8_H_10_NO_2_)]_0.4_. The molecules crystallize as layers in the *bc* plane with van der Waals interactions allowing the alkyl chains to interact and the ferrocene units to form a herringbone pattern up the *c* axis.  Every second layer is linked *via* N—H⋯O hydrogen bonding.The two complexes were modelled as disordered in a ratio of 0.60:0.40.

## Related literature

For background information, see: Barišić *et al.* (2002 ▶, 2006 ▶); Pavlović *et al.* (2003 ▶).
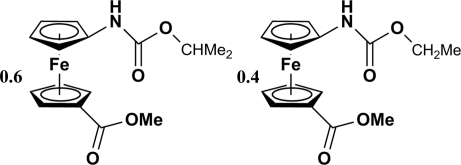


## Experimental

### 

#### Crystal data

[Fe(C_7_H_7_O_2_)(C_9_H_12_NO_2_)]_0.6_[Fe(C_7_H_7_O_2_)(C_8_H_10_NO_2_)]_0.4_
*M* *_r_* = 339.56
Monoclinic, 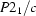

*a* = 9.7494 (19) Å
*b* = 15.624 (3) Å
*c* = 9.860 (2) Å
β = 100.82 (3)°
*V* = 1475.2 (5) Å^3^
*Z* = 4
Mo *K*α radiation
μ = 1.04 mm^−1^
*T* = 150 K
0.40 × 0.22 × 0.10 mm

#### Data collection

Nonius KappaCCD diffractometer
Absorption correction: multi-scan (*SORTAV*; Blessing, 1995 ▶) *T*_min_ = 0.740, *T*_max_ = 0.906
14609 measured reflections
3377 independent reflections
2817 reflections with *I* > 2σ(*I*)
*R*_int_ = 0.063

#### Refinement

*R*[*F* ^2^ > 2σ(*F* ^2^)] = 0.040
*wR*(*F* ^2^) = 0.100
*S* = 1.05
3377 reflections
257 parameters
391 restraints
H-atom parameters constrained
Δρ_max_ = 0.44 e Å^−3^
Δρ_min_ = −0.46 e Å^−3^

### 

Data collection: *COLLECT* (Nonius, 2001 ▶); cell refinement: *DENZO-SMN* (Otwinowski & Minor, 1997 ▶); data reduction: *DENZO-SMN*; program(s) used to solve structure: *SHELXS97* (Sheldrick, 2008 ▶); program(s) used to refine structure: *SHELXL97* (Sheldrick, 2008 ▶); molecular graphics: *SHELXTL/PC* (Sheldrick, 2008 ▶); software used to prepare material for publication: *SHELXTL/PC*.

## Supplementary Material

Crystal structure: contains datablocks global, I. DOI: 10.1107/S1600536809008204/kj2117sup1.cif

Structure factors: contains datablocks I. DOI: 10.1107/S1600536809008204/kj2117Isup2.hkl

Additional supplementary materials:  crystallographic information; 3D view; checkCIF report

## Figures and Tables

**Table 1:** Hydrogen-bond geometry (Å, °)

*D*—H⋯*A*	*D*—H	H⋯*A*	*D*⋯*A*	*D*—H⋯*A*
N15*A*—H15*A*⋯O7^i^	0.88	1.93	2.793 (14)	168
N15*B*—H15*B*⋯O7^i^	0.88	2.21	2.962 (19)	143

Symmetry code: (i) 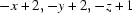.

**Fig. 1.**
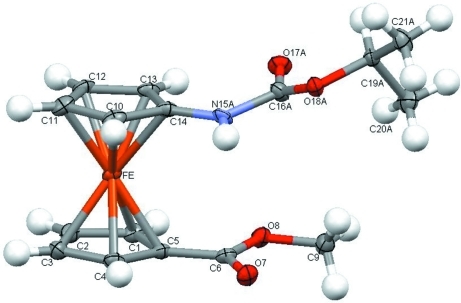
The molecular structure of the title compound with 30% probability displacement ellipsoids and the atom labelling scheme.

## supplementary crystallographic information

### Comment

Methyloxy-2'-(tertbutyloxycarbonylamino)ferrocene-1-carboxylate [{MeOC(O)Cp}Fe{CpN(H)C(O)OCMe_3_}] (where Cp = η5-C_5_H_4_) has been used as a synthon in the preparation of ferrocene amino acid (Fca) peptide conjugates. This unnatural amino acid provides a convenient route to C– or N-terminal labelling of α-amino acids and peptides in both solution and solid phase (Barišić *et al.* 2006). The C-terminal conjugation of the natural amino acid and peptides to Fca requires the removal of the *tert*-butyloxy (t-Boc) group under acidic conditions. The resultant product {MeOC(O)Cp}Fe{CpNH_2_} is highly unstable, and usually coupled *in situ* to the active ester derivative of amino acids. In our attempts to trap and crystallize this species, the title compounds (1) and (2) were produced, which might give an insight into the decomposition pathways of the t-Boc group in acidic medium. It is believed that crystallization occurred during partial decomposition and two intermediates in the stepwise decomposition were isolated crystallographhically.

There were two molecules co-crystallized in the asymmetric unit. The two molecules were crystallographically identical except for the side chain; one was –N(H)C(O)OCHMe_2_ (60%) and the other was –N(H)C(O)OCH_2_Me (40%). The molecules revealed close contacts between the hydrogen atom attached to the nitrogen and the carbonyl atoms of the adjacent molecuel. Essentially, dimers are formed *via* hydrogen bonding (Table 1).

### Experimental

The ferrocene compound {MeOC(O)Cp}Fe{CpN(H)C(O)OCMe_3_} was prepared by standard procedures reported by Rapić and coworkers (Barišić *et al.*, 2002; Pavlović *et al.*, 2003). The synthesis of the ferrocene derivative {MeOC(O)Cp}Fe{CpNH_2_} requires the removal of the t-Boc group using trifluoroacetic acid (TFA) under argon. After several minutes, the reaction progress was quenched with a base (triethyl amine). Orange plates of the mixed crystal of the title compounds [{MeOC(O)Cp}Fe{CpN(H)C(O)OCHMe_2_}] and [{MeOC(O)Cp}Fe{CpN(H)C(O)OCH_2_Me}] were grown from a concentrated methylene chloride solution by slow diffusion of hexane.

### Refinement

All H atoms were positioned geometrically and constrained as riding atoms with C—H = 1.00Å and *U*_iso_(H) = 1.2*U*_eq_(C) for methyne H atoms and C—H = 0.99Å and *U*_iso_(H) = 1.2*U*_eq_(C) for methylene H atoms and C—H = 0.98Å and *U*_iso_(H) = 1.5*U*_eq_(C) for methyl H atoms and N—H = 0.88Å and *U*_iso_(H) = 1.2*U*_eq_(C) for amine H atoms. Soft proximity (SIMU) and rigid-bond restraints (DELU) were applied to the anisotropic displacement parameters.

### Crystal data

**Table:**

[Fe(C_7_H_7_O_2_)(C_9_H_12_NO_2_)]_0.6_[Fe(C_7_H_7_O_2_)(C_8_H_10_NO_2_)]_0.4_	*F*(000) = 707
*M**_r_* = 339.56	*D*_x_ = 1.529 Mg m^−^^3^
Monoclinic, *P*2_1_/*c*	Mo *K*α radiation, λ = 0.71073 Å
Hall symbol: -P 2ybc	Cell parameters from 13419 reflections
*a* = 9.7494 (19) Å	θ = 2.0–27.5°
*b* = 15.624 (3) Å	µ = 1.04 mm^−^^1^
*c* = 9.860 (2) Å	*T* = 150 K
β = 100.82 (3)°	Plate, orange
*V* = 1475.2 (5) Å^3^	0.40 × 0.22 × 0.10 mm
*Z* = 4	

### Data collection

**Table:**

Nonius KappaCCD diffractometer	3377 independent reflections
Radiation source: fine-focus sealed tube	2817 reflections with *I* > 2σ(*I*)
graphite	*R*_int_ = 0.063
φ scans, and ω scans with κ offsets	θ_max_ = 27.5°, θ_min_ = 2.6°
Absorption correction: multi-scan from symmetry-related measurements (*SORTAV*; Blessing, 1995)	*h* = −12→12
*T*_min_ = 0.740, *T*_max_ = 0.906	*k* = −20→20
14609 measured reflections	*l* = −12→12

### Refinement

**Table:**

Refinement on *F*^2^	Primary atom site location: structure-invariant direct methods
Least-squares matrix: full	Secondary atom site location: difference Fourier map
*R*[*F*^2^ > 2σ(*F*^2^)] = 0.040	Hydrogen site location: inferred from neighbouring sites
*wR*(*F*^2^) = 0.100	H-atom parameters constrained
*S* = 1.05	*w* = 1/[σ^2^(*F*_o_^2^) + (0.0444*P*)^2^ + 1.0113*P*] where *P* = (*F*_o_^2^ + 2*F*_c_^2^)/3
3377 reflections	(Δ/σ)_max_ = 0.002
257 parameters	Δρ_max_ = 0.44 e Å^−^^3^
391 restraints	Δρ_min_ = −0.46 e Å^−^^3^

### Special details

**Table:**

Geometry. All e.s.d.'s (except the e.s.d. in the dihedral angle between two l.s. planes) are estimated using the full covariance matrix. The cell e.s.d.'s are taken into account individually in the estimation of e.s.d.'s in distances, angles and torsion angles; correlations between e.s.d.'s in cell parameters are only used when they are defined by crystal symmetry. An approximate (isotropic) treatment of cell e.s.d.'s is used for estimating e.s.d.'s involving l.s. planes.
Refinement. Refinement of *F*^2^ against ALL reflections. The weighted *R*-factor *wR* and goodness of fit *S* are based on *F*^2^, conventional *R*-factors *R* are based on *F*, with *F* set to zero for negative *F*^2^. The threshold expression of *F*^2^ > σ(*F*^2^) is used only for calculating *R*-factors(gt) *etc*. and is not relevant to the choice of reflections for refinement. *R*-factors based on *F*^2^ are statistically about twice as large as those based on *F*, and *R*- factors based on ALL data will be even larger.

### Fractional atomic coordinates and isotropic or equivalent isotropic displacement parameters (Å^2^)

**Table:**

	*x*	*y*	*z*	*U*_iso_*/*U*_eq_	Occ. (<1)
Fe	0.78558 (3)	1.126971 (18)	0.20906 (3)	0.02654 (11)	
C1	0.6804 (2)	1.19504 (13)	0.3330 (2)	0.0321 (4)	
H1A	0.5807	1.1867	0.3418	0.039*	
C2	0.7297 (3)	1.25123 (14)	0.2403 (2)	0.0367 (5)	
H2A	0.6705	1.2887	0.1706	0.044*	
C3	0.8778 (3)	1.24367 (14)	0.2608 (2)	0.0372 (5)	
H3A	0.9403	1.2750	0.2080	0.045*	
C4	0.9215 (2)	1.18269 (14)	0.3677 (2)	0.0323 (4)	
H4A	1.0197	1.1642	0.4040	0.039*	
C5	0.7997 (2)	1.15270 (13)	0.4133 (2)	0.0281 (4)	
C6	0.7987 (2)	1.08701 (13)	0.5188 (2)	0.0279 (4)	
O7	0.90170 (15)	1.05702 (10)	0.59097 (17)	0.0360 (4)	
O8	0.66914 (15)	1.06384 (10)	0.53090 (16)	0.0329 (3)	
C9	0.6609 (3)	1.00237 (16)	0.6387 (3)	0.0401 (5)	
H9A	0.7121	0.9504	0.6229	0.060*	
H9B	0.5629	0.9880	0.6377	0.060*	
H9C	0.7022	1.0270	0.7285	0.060*	
C10	0.8967 (2)	1.03436 (15)	0.1286 (2)	0.0364 (5)	
H10A	0.9987	1.0223	0.1575	0.044*	
C11	0.8348 (3)	1.09292 (16)	0.0240 (2)	0.0390 (5)	
H11A	0.8860	1.1292	−0.0336	0.047*	
C12	0.6877 (2)	1.09098 (15)	0.0158 (2)	0.0366 (5)	
H12A	0.6178	1.1258	−0.0484	0.044*	
C13	0.6566 (2)	1.03129 (14)	0.1156 (2)	0.0326 (5)	
H13A	0.5618	1.0167	0.1336	0.039*	
C14	0.7866 (2)	0.99586 (13)	0.1840 (2)	0.0308 (4)	
N15A	0.8170 (15)	0.9348 (8)	0.2832 (15)	0.0298 (18)	0.60
H15A	0.9036	0.9299	0.3281	0.036*	0.60
C16A	0.7063 (14)	0.8747 (8)	0.3191 (11)	0.034 (2)	0.60
O17A	0.5884 (19)	0.8780 (12)	0.2670 (11)	0.039 (2)	0.60
O18A	0.7847 (3)	0.80918 (19)	0.3703 (3)	0.0288 (6)	0.60
C19A	0.6961 (4)	0.7395 (3)	0.4008 (5)	0.0297 (8)	0.60
H19A	0.6203	0.7287	0.3191	0.036*	0.60
C20A	0.6332 (6)	0.7639 (4)	0.5229 (6)	0.0466 (13)	0.60
H20A	0.7078	0.7762	0.6019	0.070*	0.60
H20B	0.5759	0.7166	0.5462	0.070*	0.60
H20C	0.5747	0.8149	0.5005	0.070*	0.60
C21A	0.7913 (5)	0.6619 (3)	0.4267 (7)	0.0431 (12)	0.60
H21A	0.8368	0.6530	0.3473	0.065*	0.60
H21B	0.7360	0.6113	0.4398	0.065*	0.60
H21C	0.8624	0.6715	0.5098	0.065*	0.60
N15B	0.797 (2)	0.9282 (13)	0.289 (2)	0.032 (3)	0.40
H15B	0.8683	0.9303	0.3582	0.038*	0.40
C16B	0.7224 (19)	0.8744 (13)	0.2867 (15)	0.024 (2)	0.40
O17B	0.595 (3)	0.8684 (18)	0.2333 (18)	0.039 (3)	0.40
O18B	0.7611 (6)	0.8251 (3)	0.4349 (6)	0.0426 (12)	0.40
C19B	0.6752 (9)	0.7564 (6)	0.4736 (12)	0.052 (2)	0.40
H19B	0.5754	0.7688	0.4374	0.062*	0.40
H19C	0.6887	0.7526	0.5755	0.062*	0.40
C20B	0.7132 (13)	0.6753 (6)	0.4178 (12)	0.078 (3)	0.40
H20D	0.6956	0.6784	0.3167	0.117*	0.40
H20E	0.6570	0.6292	0.4469	0.117*	0.40
H20F	0.8124	0.6638	0.4522	0.117*	0.40

### Atomic displacement parameters (Å^2^)

**Table:**

	*U*^11^	*U*^22^	*U*^33^	*U*^12^	*U*^13^	*U*^23^
Fe	0.02407 (16)	0.03112 (17)	0.02215 (17)	0.00247 (11)	−0.00153 (11)	−0.00056 (11)
C1	0.0310 (10)	0.0354 (11)	0.0288 (11)	0.0068 (8)	0.0026 (9)	−0.0032 (8)
C2	0.0463 (13)	0.0294 (10)	0.0315 (12)	0.0075 (9)	−0.0005 (10)	−0.0002 (9)
C3	0.0430 (12)	0.0326 (11)	0.0338 (12)	−0.0065 (9)	0.0012 (10)	0.0016 (9)
C4	0.0280 (10)	0.0373 (11)	0.0286 (11)	−0.0048 (8)	−0.0024 (8)	−0.0038 (9)
C5	0.0295 (10)	0.0305 (9)	0.0222 (10)	0.0007 (8)	−0.0008 (8)	−0.0044 (8)
C6	0.0258 (10)	0.0331 (10)	0.0229 (10)	0.0007 (8)	−0.0004 (8)	−0.0056 (8)
O7	0.0279 (8)	0.0424 (9)	0.0335 (9)	0.0016 (6)	−0.0052 (6)	0.0066 (7)
O8	0.0255 (7)	0.0429 (8)	0.0287 (8)	−0.0008 (6)	0.0008 (6)	0.0046 (6)
C9	0.0379 (12)	0.0491 (13)	0.0325 (12)	−0.0051 (10)	0.0045 (10)	0.0053 (10)
C10	0.0332 (11)	0.0426 (12)	0.0324 (12)	0.0082 (9)	0.0042 (9)	−0.0071 (9)
C11	0.0418 (13)	0.0499 (13)	0.0255 (11)	0.0050 (10)	0.0066 (9)	−0.0041 (9)
C12	0.0390 (12)	0.0428 (12)	0.0228 (10)	0.0048 (9)	−0.0074 (9)	−0.0022 (9)
C13	0.0283 (10)	0.0349 (11)	0.0298 (11)	0.0012 (8)	−0.0072 (8)	−0.0048 (9)
C14	0.0284 (10)	0.0302 (10)	0.0291 (11)	0.0031 (8)	−0.0068 (9)	−0.0061 (8)
N15A	0.022 (4)	0.027 (2)	0.036 (3)	−0.010 (2)	−0.005 (3)	−0.003 (2)
C16A	0.033 (4)	0.027 (2)	0.040 (6)	−0.004 (2)	−0.004 (3)	0.003 (3)
O17A	0.027 (2)	0.045 (4)	0.040 (5)	−0.002 (2)	−0.011 (4)	0.000 (4)
O18A	0.0233 (13)	0.0283 (14)	0.0325 (17)	−0.0025 (10)	−0.0009 (13)	0.0076 (13)
C19A	0.032 (2)	0.028 (2)	0.027 (2)	−0.0064 (14)	0.0018 (17)	0.0043 (16)
C20A	0.044 (3)	0.056 (3)	0.044 (3)	−0.010 (2)	0.021 (2)	0.000 (2)
C21A	0.040 (2)	0.031 (2)	0.055 (3)	0.0009 (19)	0.000 (2)	0.0122 (19)
N15B	0.013 (5)	0.030 (5)	0.047 (5)	0.004 (3)	−0.012 (3)	0.008 (4)
C16B	0.017 (4)	0.037 (4)	0.017 (5)	0.002 (3)	0.001 (3)	0.006 (3)
O17B	0.031 (4)	0.048 (6)	0.032 (7)	−0.006 (3)	−0.008 (5)	−0.002 (5)
O18B	0.038 (3)	0.038 (3)	0.045 (3)	−0.007 (2)	−0.009 (2)	0.014 (2)
C19B	0.045 (5)	0.054 (5)	0.057 (7)	−0.010 (4)	0.012 (4)	0.023 (4)
C20B	0.115 (9)	0.047 (5)	0.069 (6)	−0.020 (6)	0.011 (7)	0.004 (4)

### Geometric parameters (Å, °)

**Table:**

Fe—C5	2.033 (2)	C12—C13	1.429 (3)
Fe—C1	2.037 (2)	C12—H12A	1.0000
Fe—C11	2.042 (2)	C13—C14	1.431 (3)
Fe—C12	2.043 (2)	C13—H13A	1.0000
Fe—C4	2.045 (2)	C14—N15A	1.358 (13)
Fe—C10	2.054 (2)	C14—N15B	1.467 (18)
Fe—C3	2.055 (2)	N15A—C16A	1.522 (17)
Fe—C2	2.055 (2)	N15A—H15A	0.8800
Fe—C13	2.057 (2)	C16A—O17A	1.17 (2)
Fe—C14	2.064 (2)	C16A—O18A	1.320 (14)
C1—C2	1.415 (3)	O18A—C19A	1.455 (5)
C1—C5	1.439 (3)	C19A—C20A	1.500 (6)
C1—H1A	1.0000	C19A—C21A	1.518 (6)
C2—C3	1.425 (3)	C19A—H19A	1.0000
C2—H2A	1.0000	C20A—H20A	0.9800
C3—C4	1.425 (3)	C20A—H20B	0.9800
C3—H3A	1.0000	C20A—H20C	0.9800
C4—C5	1.426 (3)	C21A—H21A	0.9800
C4—H4A	1.0000	C21A—H21B	0.9800
C5—C6	1.463 (3)	C21A—H21C	0.9800
C6—O7	1.211 (2)	N15B—C16B	1.11 (3)
C6—O8	1.340 (2)	N15B—H15B	0.8800
O8—C9	1.446 (3)	C16B—O17B	1.26 (3)
C9—H9A	0.9800	C16B—O18B	1.632 (16)
C9—H9B	0.9800	O18B—C19B	1.455 (9)
C9—H9C	0.9800	C19B—C20B	1.457 (15)
C10—C11	1.425 (3)	C19B—H19B	0.9900
C10—C14	1.425 (3)	C19B—H19C	0.9900
C10—H10A	1.0000	C20B—H20D	0.9800
C11—C12	1.421 (3)	C20B—H20E	0.9800
C11—H11A	1.0000	C20B—H20F	0.9800
			
C5—Fe—C1	41.42 (8)	O7—C6—O8	122.3 (2)
C5—Fe—C11	162.38 (9)	O7—C6—C5	125.03 (19)
C1—Fe—C11	154.35 (9)	O8—C6—C5	112.62 (17)
C5—Fe—C12	155.67 (10)	C6—O8—C9	115.33 (17)
C1—Fe—C12	120.25 (9)	O8—C9—H9A	109.5
C11—Fe—C12	40.72 (10)	O8—C9—H9B	109.5
C5—Fe—C4	40.93 (9)	H9A—C9—H9B	109.5
C1—Fe—C4	69.25 (9)	O8—C9—H9C	109.5
C11—Fe—C4	124.55 (10)	H9A—C9—H9C	109.5
C12—Fe—C4	162.28 (10)	H9B—C9—H9C	109.5
C5—Fe—C10	125.42 (9)	C11—C10—C14	107.5 (2)
C1—Fe—C10	163.99 (9)	C11—C10—Fe	69.20 (13)
C11—Fe—C10	40.71 (9)	C14—C10—Fe	70.10 (12)
C12—Fe—C10	68.51 (9)	C11—C10—H10A	126.2
C4—Fe—C10	106.41 (9)	C14—C10—H10A	126.2
C5—Fe—C3	68.50 (9)	Fe—C10—H10A	126.2
C1—Fe—C3	68.52 (10)	C12—C11—C10	108.2 (2)
C11—Fe—C3	106.72 (10)	C12—C11—Fe	69.68 (13)
C12—Fe—C3	125.58 (10)	C10—C11—Fe	70.09 (13)
C4—Fe—C3	40.69 (9)	C12—C11—H11A	125.9
C10—Fe—C3	118.89 (10)	C10—C11—H11A	125.9
C5—Fe—C2	68.59 (9)	Fe—C11—H11A	125.9
C1—Fe—C2	40.46 (9)	C11—C12—C13	108.5 (2)
C11—Fe—C2	119.57 (10)	C11—C12—Fe	69.60 (13)
C12—Fe—C2	108.02 (9)	C13—C12—Fe	70.12 (12)
C4—Fe—C2	68.59 (9)	C11—C12—H12A	125.8
C10—Fe—C2	153.80 (10)	C13—C12—H12A	125.8
C3—Fe—C2	40.57 (9)	Fe—C12—H12A	125.8
C5—Fe—C13	120.63 (9)	C12—C13—C14	107.0 (2)
C1—Fe—C13	108.29 (9)	C12—C13—Fe	69.08 (12)
C11—Fe—C13	68.71 (10)	C14—C13—Fe	69.93 (12)
C12—Fe—C13	40.80 (9)	C12—C13—H13A	126.5
C4—Fe—C13	154.95 (9)	C14—C13—H13A	126.5
C10—Fe—C13	68.74 (9)	Fe—C13—H13A	126.5
C3—Fe—C13	163.45 (9)	N15A—C14—C10	119.6 (6)
C2—Fe—C13	126.64 (9)	N15A—C14—C13	131.7 (6)
C5—Fe—C14	108.30 (9)	C10—C14—C13	108.7 (2)
C1—Fe—C14	127.19 (9)	C10—C14—N15B	128.0 (8)
C11—Fe—C14	68.11 (10)	C13—C14—N15B	123.3 (8)
C12—Fe—C14	68.11 (9)	N15A—C14—Fe	128.0 (6)
C4—Fe—C14	119.81 (9)	C10—C14—Fe	69.39 (12)
C10—Fe—C14	40.51 (9)	C13—C14—Fe	69.43 (12)
C3—Fe—C14	154.02 (9)	N15B—C14—Fe	129.3 (10)
C2—Fe—C14	164.38 (9)	C14—N15A—C16A	122.2 (11)
C13—Fe—C14	40.65 (8)	C14—N15A—H15A	118.9
C2—C1—C5	107.62 (19)	C16A—N15A—H15A	118.9
C2—C1—Fe	70.47 (13)	O17A—C16A—O18A	130.7 (14)
C5—C1—Fe	69.14 (12)	O17A—C16A—N15A	123.6 (14)
C2—C1—H1A	126.2	O18A—C16A—N15A	100.6 (10)
C5—C1—H1A	126.2	C16A—O18A—C19A	109.7 (6)
Fe—C1—H1A	126.2	O18A—C19A—C20A	108.9 (4)
C1—C2—C3	108.39 (19)	O18A—C19A—C21A	105.3 (4)
C1—C2—Fe	69.07 (12)	C20A—C19A—C21A	113.4 (4)
C3—C2—Fe	69.68 (12)	O18A—C19A—H19A	109.7
C1—C2—H2A	125.8	C20A—C19A—H19A	109.7
C3—C2—H2A	125.8	C21A—C19A—H19A	109.7
Fe—C2—H2A	125.8	C16B—N15B—C14	125.1 (19)
C2—C3—C4	108.3 (2)	C16B—N15B—H15B	117.5
C2—C3—Fe	69.74 (12)	C14—N15B—H15B	117.5
C4—C3—Fe	69.29 (12)	N15B—C16B—O17B	131 (2)
C2—C3—H3A	125.8	N15B—C16B—O18B	107.6 (17)
C4—C3—H3A	125.8	O17B—C16B—O18B	112.6 (15)
Fe—C3—H3A	125.8	C19B—O18B—C16B	122.3 (9)
C3—C4—C5	107.58 (19)	O18B—C19B—C20B	110.0 (9)
C3—C4—Fe	70.02 (13)	O18B—C19B—H19B	109.7
C5—C4—Fe	69.08 (12)	C20B—C19B—H19B	109.7
C3—C4—H4A	126.2	O18B—C19B—H19C	109.7
C5—C4—H4A	126.2	C20B—C19B—H19C	109.7
Fe—C4—H4A	126.2	H19B—C19B—H19C	108.2
C4—C5—C1	108.09 (19)	C19B—C20B—H20D	109.5
C4—C5—C6	124.89 (19)	C19B—C20B—H20E	109.5
C1—C5—C6	126.95 (19)	H20D—C20B—H20E	109.5
C4—C5—Fe	69.99 (12)	C19B—C20B—H20F	109.5
C1—C5—Fe	69.44 (12)	H20D—C20B—H20F	109.5
C6—C5—Fe	123.90 (14)	H20E—C20B—H20F	109.5
			
C5—Fe—C1—C2	118.61 (18)	C4—Fe—C10—C14	116.94 (13)
C11—Fe—C1—C2	−46.8 (3)	C3—Fe—C10—C14	159.18 (12)
C12—Fe—C1—C2	−82.43 (16)	C2—Fe—C10—C14	−168.33 (18)
C4—Fe—C1—C2	80.95 (14)	C13—Fe—C10—C14	−37.06 (13)
C10—Fe—C1—C2	157.9 (3)	C14—C10—C11—C12	0.4 (3)
C3—Fe—C1—C2	37.20 (13)	Fe—C10—C11—C12	−59.44 (16)
C13—Fe—C1—C2	−125.52 (14)	C14—C10—C11—Fe	59.87 (15)
C14—Fe—C1—C2	−166.73 (13)	C5—Fe—C11—C12	163.0 (3)
C11—Fe—C1—C5	−165.4 (2)	C1—Fe—C11—C12	−50.5 (3)
C12—Fe—C1—C5	158.97 (13)	C4—Fe—C11—C12	−166.63 (13)
C4—Fe—C1—C5	−37.65 (12)	C10—Fe—C11—C12	119.3 (2)
C10—Fe—C1—C5	39.3 (4)	C3—Fe—C11—C12	−125.60 (15)
C3—Fe—C1—C5	−81.41 (14)	C2—Fe—C11—C12	−83.45 (16)
C2—Fe—C1—C5	−118.61 (18)	C13—Fe—C11—C12	37.56 (13)
C13—Fe—C1—C5	115.87 (13)	C14—Fe—C11—C12	81.42 (15)
C14—Fe—C1—C5	74.67 (15)	C5—Fe—C11—C10	43.7 (4)
C5—C1—C2—C3	0.7 (2)	C1—Fe—C11—C10	−169.82 (19)
Fe—C1—C2—C3	−58.74 (16)	C12—Fe—C11—C10	−119.3 (2)
C5—C1—C2—Fe	59.40 (14)	C4—Fe—C11—C10	74.08 (17)
C5—Fe—C2—C1	−38.60 (13)	C3—Fe—C11—C10	115.10 (15)
C11—Fe—C2—C1	158.74 (13)	C2—Fe—C11—C10	157.25 (14)
C12—Fe—C2—C1	115.77 (14)	C13—Fe—C11—C10	−81.73 (15)
C4—Fe—C2—C1	−82.72 (14)	C14—Fe—C11—C10	−37.87 (14)
C10—Fe—C2—C1	−166.43 (19)	C10—C11—C12—C13	0.2 (3)
C3—Fe—C2—C1	−120.12 (19)	Fe—C11—C12—C13	−59.54 (16)
C13—Fe—C2—C1	74.38 (16)	C10—C11—C12—Fe	59.71 (16)
C14—Fe—C2—C1	42.8 (4)	C5—Fe—C12—C11	−167.56 (19)
C5—Fe—C2—C3	81.52 (14)	C1—Fe—C12—C11	157.25 (14)
C1—Fe—C2—C3	120.12 (19)	C4—Fe—C12—C11	38.7 (4)
C11—Fe—C2—C3	−81.14 (16)	C10—Fe—C12—C11	−37.68 (14)
C12—Fe—C2—C3	−124.11 (14)	C3—Fe—C12—C11	73.22 (17)
C4—Fe—C2—C3	37.40 (14)	C2—Fe—C12—C11	114.68 (15)
C10—Fe—C2—C3	−46.3 (3)	C13—Fe—C12—C11	−119.62 (19)
C13—Fe—C2—C3	−165.50 (14)	C14—Fe—C12—C11	−81.43 (15)
C14—Fe—C2—C3	162.9 (3)	C5—Fe—C12—C13	−47.9 (3)
C1—C2—C3—C4	−0.4 (3)	C1—Fe—C12—C13	−83.13 (15)
Fe—C2—C3—C4	−58.72 (15)	C11—Fe—C12—C13	119.62 (19)
C1—C2—C3—Fe	58.36 (15)	C4—Fe—C12—C13	158.4 (3)
C5—Fe—C3—C2	−81.77 (15)	C10—Fe—C12—C13	81.94 (14)
C1—Fe—C3—C2	−37.10 (14)	C3—Fe—C12—C13	−167.16 (13)
C11—Fe—C3—C2	116.20 (15)	C2—Fe—C12—C13	−125.70 (14)
C12—Fe—C3—C2	75.49 (17)	C14—Fe—C12—C13	38.19 (13)
C4—Fe—C3—C2	−119.8 (2)	C11—C12—C13—C14	−0.7 (2)
C10—Fe—C3—C2	158.62 (14)	Fe—C12—C13—C14	−59.91 (15)
C13—Fe—C3—C2	44.9 (4)	C11—C12—C13—Fe	59.22 (16)
C14—Fe—C3—C2	−169.58 (19)	C5—Fe—C13—C12	159.17 (13)
C5—Fe—C3—C4	38.06 (13)	C1—Fe—C13—C12	115.40 (14)
C1—Fe—C3—C4	82.74 (14)	C11—Fe—C13—C12	−37.49 (14)
C11—Fe—C3—C4	−123.97 (14)	C4—Fe—C13—C12	−164.6 (2)
C12—Fe—C3—C4	−164.67 (13)	C10—Fe—C13—C12	−81.33 (15)
C10—Fe—C3—C4	−81.55 (16)	C3—Fe—C13—C12	39.4 (4)
C2—Fe—C3—C4	119.8 (2)	C2—Fe—C13—C12	74.24 (17)
C13—Fe—C3—C4	164.7 (3)	C14—Fe—C13—C12	−118.27 (19)
C14—Fe—C3—C4	−49.7 (3)	C5—Fe—C13—C14	−82.56 (15)
C2—C3—C4—C5	−0.1 (2)	C1—Fe—C13—C14	−126.33 (14)
Fe—C3—C4—C5	−59.08 (14)	C11—Fe—C13—C14	80.78 (15)
C2—C3—C4—Fe	59.00 (15)	C12—Fe—C13—C14	118.27 (19)
C5—Fe—C4—C3	−118.89 (19)	C4—Fe—C13—C14	−46.4 (3)
C1—Fe—C4—C3	−80.80 (15)	C10—Fe—C13—C14	36.94 (14)
C11—Fe—C4—C3	74.64 (17)	C3—Fe—C13—C14	157.6 (3)
C12—Fe—C4—C3	44.9 (3)	C2—Fe—C13—C14	−167.49 (13)
C10—Fe—C4—C3	115.47 (15)	C11—C10—C14—N15A	177.8 (7)
C2—Fe—C4—C3	−37.30 (14)	Fe—C10—C14—N15A	−122.9 (7)
C13—Fe—C4—C3	−169.8 (2)	C11—C10—C14—C13	−0.9 (2)
C14—Fe—C4—C3	157.33 (14)	Fe—C10—C14—C13	58.44 (15)
C1—Fe—C4—C5	38.09 (12)	C11—C10—C14—N15B	176.4 (12)
C11—Fe—C4—C5	−166.47 (13)	Fe—C10—C14—N15B	−124.3 (13)
C12—Fe—C4—C5	163.8 (3)	C11—C10—C14—Fe	−59.30 (15)
C10—Fe—C4—C5	−125.64 (13)	C12—C13—C14—N15A	−177.5 (9)
C3—Fe—C4—C5	118.89 (19)	Fe—C13—C14—N15A	123.2 (9)
C2—Fe—C4—C5	81.58 (14)	C12—C13—C14—C10	0.9 (2)
C13—Fe—C4—C5	−50.9 (3)	Fe—C13—C14—C10	−58.42 (15)
C14—Fe—C4—C5	−83.78 (14)	C12—C13—C14—N15B	−176.4 (12)
C3—C4—C5—C1	0.5 (2)	Fe—C13—C14—N15B	124.2 (12)
Fe—C4—C5—C1	−59.19 (14)	C12—C13—C14—Fe	59.37 (15)
C3—C4—C5—C6	177.69 (19)	C5—Fe—C14—N15A	−11.5 (8)
Fe—C4—C5—C6	118.0 (2)	C1—Fe—C14—N15A	−53.7 (8)
C3—C4—C5—Fe	59.68 (15)	C11—Fe—C14—N15A	150.1 (8)
C2—C1—C5—C4	−0.7 (2)	C12—Fe—C14—N15A	−165.8 (8)
Fe—C1—C5—C4	59.53 (15)	C4—Fe—C14—N15A	31.8 (8)
C2—C1—C5—C6	−177.8 (2)	C10—Fe—C14—N15A	112.1 (8)
Fe—C1—C5—C6	−117.6 (2)	C3—Fe—C14—N15A	66.8 (8)
C2—C1—C5—Fe	−60.24 (15)	C2—Fe—C14—N15A	−87.3 (8)
C1—Fe—C5—C4	−119.31 (18)	C13—Fe—C14—N15A	−127.5 (8)
C11—Fe—C5—C4	39.5 (4)	C5—Fe—C14—C10	−123.56 (14)
C12—Fe—C5—C4	−168.1 (2)	C1—Fe—C14—C10	−165.78 (13)
C10—Fe—C5—C4	73.07 (16)	C11—Fe—C14—C10	38.06 (14)
C3—Fe—C5—C4	−37.84 (13)	C12—Fe—C14—C10	82.10 (15)
C2—Fe—C5—C4	−81.59 (14)	C4—Fe—C14—C10	−80.25 (15)
C13—Fe—C5—C4	157.56 (12)	C3—Fe—C14—C10	−45.3 (3)
C14—Fe—C5—C4	114.70 (13)	C2—Fe—C14—C10	160.6 (3)
C11—Fe—C5—C1	158.8 (3)	C13—Fe—C14—C10	120.42 (19)
C12—Fe—C5—C1	−48.8 (3)	C5—Fe—C14—C13	116.01 (14)
C4—Fe—C5—C1	119.31 (18)	C1—Fe—C14—C13	73.79 (16)
C10—Fe—C5—C1	−167.61 (13)	C11—Fe—C14—C13	−82.37 (15)
C3—Fe—C5—C1	81.47 (14)	C12—Fe—C14—C13	−38.33 (14)
C2—Fe—C5—C1	37.73 (13)	C4—Fe—C14—C13	159.33 (13)
C13—Fe—C5—C1	−83.12 (14)	C10—Fe—C14—C13	−120.42 (19)
C14—Fe—C5—C1	−125.99 (13)	C3—Fe—C14—C13	−165.7 (2)
C1—Fe—C5—C6	121.4 (2)	C2—Fe—C14—C13	40.2 (4)
C11—Fe—C5—C6	−79.7 (4)	C5—Fe—C14—N15B	−0.8 (10)
C12—Fe—C5—C6	72.6 (3)	C1—Fe—C14—N15B	−43.0 (10)
C4—Fe—C5—C6	−119.3 (2)	C11—Fe—C14—N15B	160.8 (10)
C10—Fe—C5—C6	−46.2 (2)	C12—Fe—C14—N15B	−155.1 (10)
C3—Fe—C5—C6	−157.1 (2)	C4—Fe—C14—N15B	42.5 (10)
C2—Fe—C5—C6	159.2 (2)	C10—Fe—C14—N15B	122.8 (10)
C13—Fe—C5—C6	38.3 (2)	C3—Fe—C14—N15B	77.5 (10)
C14—Fe—C5—C6	−4.56 (19)	C2—Fe—C14—N15B	−76.6 (11)
C4—C5—C6—O7	8.7 (3)	C13—Fe—C14—N15B	−116.8 (10)
C1—C5—C6—O7	−174.7 (2)	C10—C14—N15A—C16A	−163.1 (9)
Fe—C5—C6—O7	96.7 (2)	C13—C14—N15A—C16A	15.1 (18)
C4—C5—C6—O8	−172.83 (19)	N15B—C14—N15A—C16A	9(10)
C1—C5—C6—O8	3.8 (3)	Fe—C14—N15A—C16A	110.8 (11)
Fe—C5—C6—O8	−84.8 (2)	C14—N15A—C16A—O17A	−2(2)
O7—C6—O8—C9	1.7 (3)	C14—N15A—C16A—O18A	155.1 (11)
C5—C6—O8—C9	−176.83 (18)	O17A—C16A—O18A—C19A	−20.4 (14)
C5—Fe—C10—C11	−165.14 (14)	N15A—C16A—O18A—C19A	−174.9 (7)
C1—Fe—C10—C11	163.9 (3)	C16A—O18A—C19A—C20A	−70.8 (7)
C12—Fe—C10—C11	37.69 (15)	C16A—O18A—C19A—C21A	167.3 (6)
C4—Fe—C10—C11	−124.34 (15)	N15A—C14—N15B—C16B	−149 (13)
C3—Fe—C10—C11	−82.10 (16)	C10—C14—N15B—C16B	−140.6 (19)
C2—Fe—C10—C11	−49.6 (3)	C13—C14—N15B—C16B	36 (3)
C13—Fe—C10—C11	81.65 (15)	Fe—C14—N15B—C16B	126 (2)
C14—Fe—C10—C11	118.7 (2)	C14—N15B—C16B—O17B	−27 (4)
C5—Fe—C10—C14	76.14 (15)	C14—N15B—C16B—O18B	−170.7 (18)
C1—Fe—C10—C14	45.2 (4)	N15B—C16B—O18B—C19B	173.6 (16)
C11—Fe—C10—C14	−118.7 (2)	O17B—C16B—O18B—C19B	22 (2)
C12—Fe—C10—C14	−81.02 (14)	C16B—O18B—C19B—C20B	84.4 (11)

### Hydrogen-bond geometry (Å, °)

**Table:**

*D*—H···*A*	*D*—H	H···*A*	*D*···*A*	*D*—H···*A*
N15A—H15A···O7^i^	0.88	1.93	2.793 (14)	168
N15B—H15B···O7^i^	0.88	2.21	2.962 (19)	143

Symmetry codes: (i) −*x*+2, −*y*+2, −*z*+1.
